# Signet ring cell carcinoma of rectum metastasizing to synchronous renal cell carcinoma: a case report

**DOI:** 10.1186/s13256-021-02749-x

**Published:** 2021-03-18

**Authors:** Blagica Krsteska, Rubens Jovanovic, Aleksandar Eftimov, Boro Ilievski, Dragan Hadzi-Mancev, Bujar Osmani, Slavica Kostadinova-Kunovska

**Affiliations:** 1 Institute of Pathology, Faculty of Medicine, Ss. Cyril and Methodius University in Skopje, Skopje, North Macedonia; 2 Faculty of Medicine, , University Clinic of Abdominal Surgery–Ss. Cyril and Methodius University in Skopje , Skopje, North Macedonia

**Keywords:** Case report, Rectal, Signet ring cell carcinoma, Tumor to tumor metastasis, Renal cell carcinoma

## Abstract

**Background:**

Rectal signet ring cell carcinoma is a rare type of colorectal adenocarcinoma characterized by an aggressive biological behavior and poor prognosis. The co-occurrence of colorectal carcinoma and renal cell carcinoma (RCC) has found in many hundreds of patients, many of whom also have additional malignancies. Cancer to cancer metastasis is rare and an uncommon phenomenon in malignancy, especially at the time of initial diagnosis, suggesting a genetic susceptibility.

**Case presentation:**

We present the case of a 66-year-old Macedonian man with synchronous rectal signet ring cell carcinoma and RCC with tumor to tumor metastasis feature. He underwent a left nephrectomy and anterior rectal resection after complaining of constipation for 3–4 months and the appearance of synchronous tumors on the imaging studies. Morphology and immunohistochemical analysis of specimens from the RCC revealed signet ring cells identical to the rectal signet ring cell carcinoma. The next-generation sequencing study revealed mutations in TP53 and ERBB2, and microsatellite stable signet ring cell carcinoma was determined by deoxyribonucleic acid (DNA) sequencing.

**Conclusions:**

Cancer to cancer metastasis, although rare, needs to be considered in synchronous tumors. RCC, when diagnosed in multiple synchronous tumors, should be examined carefully. The paucity of reported cases indicates the need for advanced research in imaging methods for metastasis and new therapeutic approaches.

## Background

Primary signet ring cell carcinoma of the rectum (PSRCCR) is a rare variant of colorectal adenocarcinoma (AC) with an incidence of less than 1% [1]. It is diagnosed on the basis of the tumor containing > 50% of cells that contain prominent intracytoplasmic mucin with marked nuclear displacement and molding (signet ring cells). The most common site of such tumors is the stomach with the characteristic feature of linitis plastica. Presentation usually occurs at an advanced stage, and the tumor has a distinctive molecular pattern and poor prognosis [2, 3].

Reported data suggest a rare but strong association between gastrointestinal and urogenital tumors based on synchronous appearance. Cancer to cancer metastasis is defined as metastasis in histologically separate carcinomas. The synchronous occurrence of colorectal AC with renal cell carcinoma (RCC) is a rare development, with an incidence of 0.4–4.8%, but AC to RCC metastasis at initial diagnosis has been reported in fewer than five cases. To our knowledge, we report here the first reported case of RSRCCR with metastasis to RCC.

## Case presentation

**Fig. 1:**
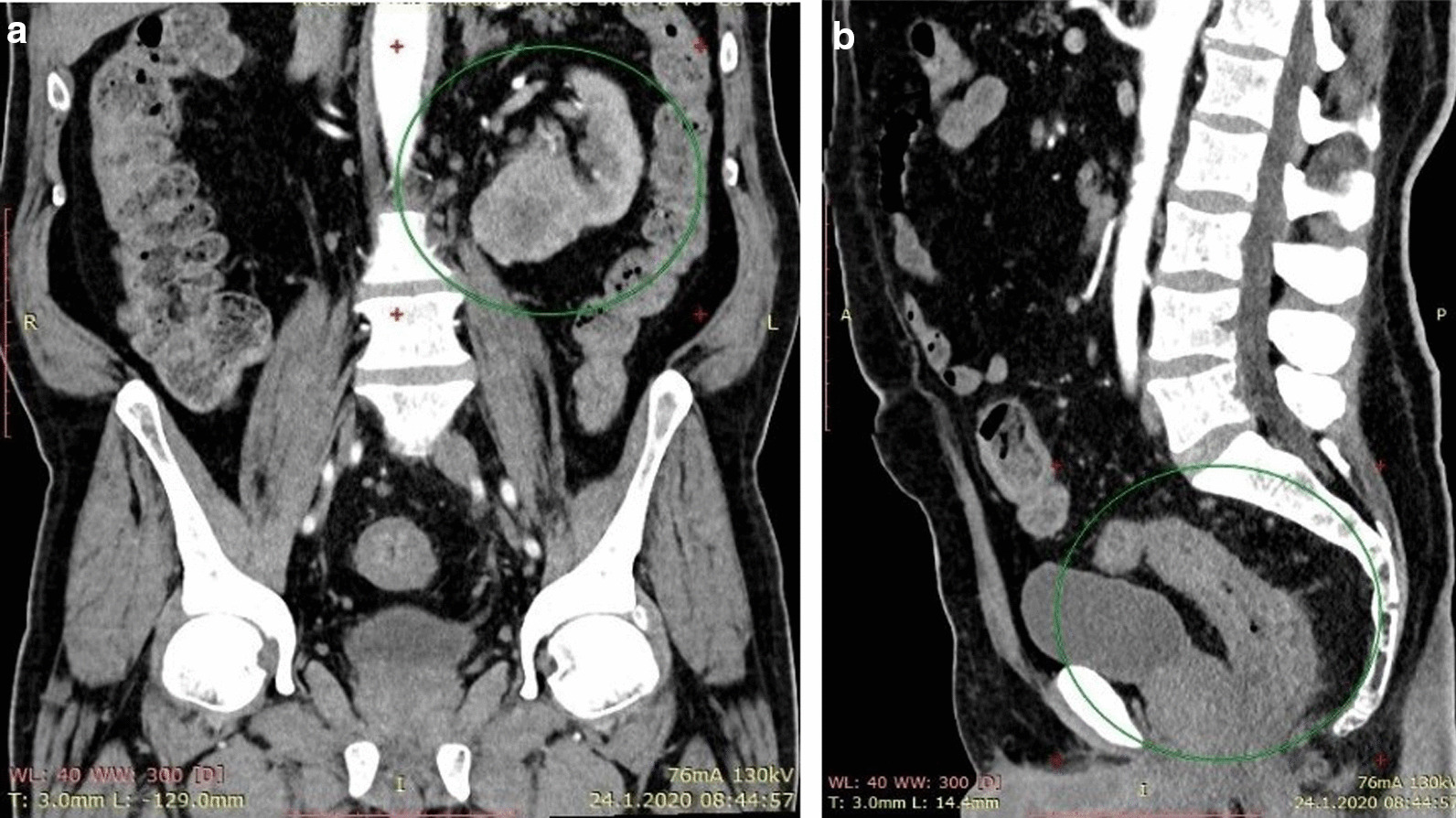
Computed tomography scan showing synchronous tumors (within green circles). **a** Kidney tumor in lower pole, **b** Rectal tumor mass with luminal narrowing

A 66-year-old Macedonian male presented with symptoms of constipation and blood in the stool (hematochezia) for 3–4 months before seeing a gastroenterologist. Review of his medical records revealed no family history of note. The outer anal examination revealed old thrombosed hemorrhoids, and endoscopy revealed an obstructive neoplastic mass located 5–7 cm from the anus. The tumor tissue showed diffuse ulcerations and bled when touched. Eight biopsy specimens were taken for pathohistological examination. The diagnosis of mucinous AC with signet ring cells was made. Radiographic examination showed no pathological findings in the liver, pancreas, spleen and lungs, but revealed a tumor mass in the left lower kidney pole with infiltrating border that caused compression to the collector system. Preoperative computed tomography confirmed synchronous tumors in the kidney and rectum (Fig. 1). The patient was admitted to the University Clinic of Abdominal Surgery in Skopje for surgical treatment. Laboratory tests showed elevated values for the enzymes lactate dehydrogenase (612 U/L), alkaline phosphatase (387 U/L) and C-reactive protein (up to 45.7 mg/L). A medial laparotomy was performed, with surgical resection of the anterior rectal area, simultaneously with left nephrectomy. Carcinosis in the small pelvis was observed.

**Fig. 2:**
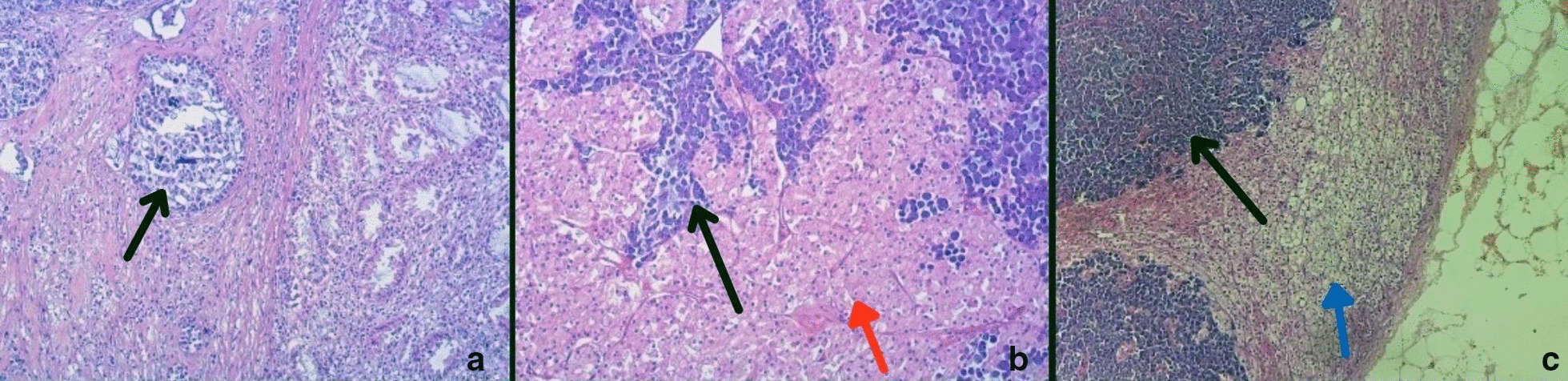
Microscropic images of signet ring cell carcinoma (hematoxylin & eosin stain). Black arrow indicates signet ring cells **a** Primary tumor in rectum (magnification: × 100). **b** Metastasis in renal cell carcinoma (RCC); red arrow indicates RCC (magnification: × 100). **c** Metastasis in adrenal gland; blue arrow indicates metastasis in adrenal gland (Magnification: × 50). Black arrow indicates signet ring cells, red arrow indicates RCC and blue arrow indicates adrenal gland

**Fig. 3:**
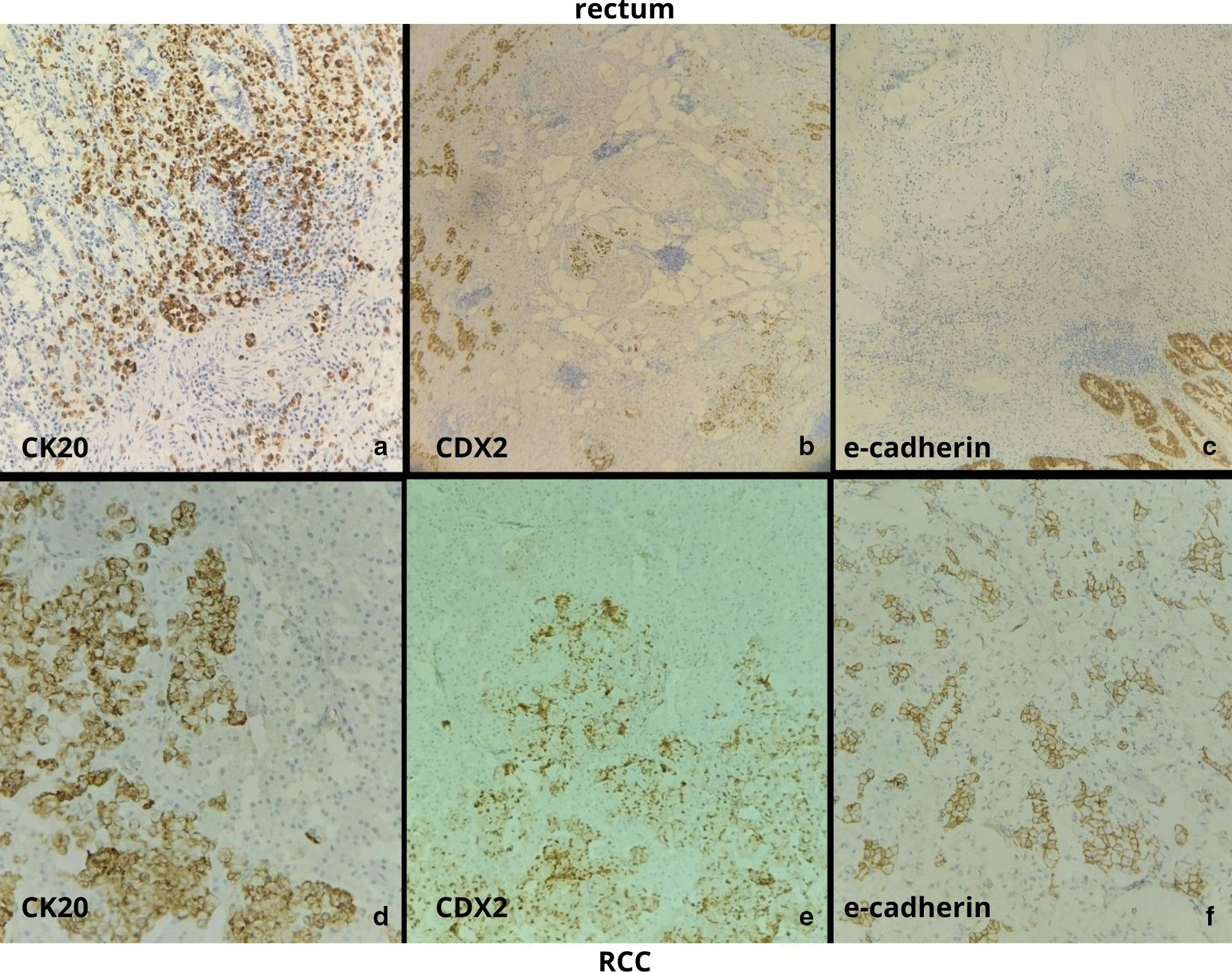
Immunohistochemistry results showing identical expression in the primary and metastatic components of the signet ring cells in RCC. **a** CK20 rectum (magnification: × 100). **b** CDX-2 rectum (magnification: × 50). **c** E-cadherin rectum (magnification: × 100). **d** CK20 RCC (magnification: × 100). **e** CDX-2 RCC (magnification: × 100). **f** E-cadherin RCC (magnification: × 200)

Tissue samples were analyzed at the Institute of pathology, Faculty of Medicine in Skopje. Gross rectal examination of tissue specimens showed a rectal tumor measuring 5.5 cm infiltrating into the perirectal fat. The kidney contained a yellowish round tumor measuring 5 cm, with necrosis and hemorrhage. The adrenal gland was slightly enlarged into perirenal fat. The tissue specimens were fixed in formalin, embedded in Paraffin and routinely stained with hematoxylin & eosin stain. Microscopic analysis revealed PSRCCR with nodal metastasis, lymphatic and vascular tumor emboli and uncommon metastasis to synchronous RCC and to the adrenal gland (Fig. 2). The tumor was classified as Stage IV according to the pTNM/UICC staging system. All specimens were analyzed immunohistochemicaly with CK20, CDX2, vimentin, RCC, E-cadherin and the mismatch repair (MMR) proteins MLH1, MSH2, MSH6 and PMS2. Signet ring cells were positive for CDX2 and CK20 (Table 1). The absence of expression of E-cadherin in the metastasized cells indicated that they were the same as those in the primary carcinoma (Fig. 3).

**Table 1:** Immunohistochemical analysis of renal cell carcinoma, rectal signet ring cell carcinoma and signet ring cell component in renal cell carcinoma

Antibody	RCC	Signet ring cell carcinoma	Signet ring cells in metastatic tumor
CK20	−	+	+
CDX2	−	+	+
Vimentin	+	−	−
E-Cadherin	+	−	−
RCC	+	−	−
MLH1	/	/	Loss of nuclear signal
MSH2	/	/	Loss of nuclear signal
PMS2	/	/	Loss of nuclear signal
MSH6	/	/	Nuclear expression

*RCC* Renal cell carcinoma, *CK20* Cytokeratin 20, *CDX2* Caudal-type homebox 2, *RCC* Renal cell carcinoma, *MLH1* MutL homolog 1, *MSH2* MutS homolog 2, *PMS2* Postmeiotic Segregation Increased 2, *MSH6* MutS homolog 6

**Fig. 4:**
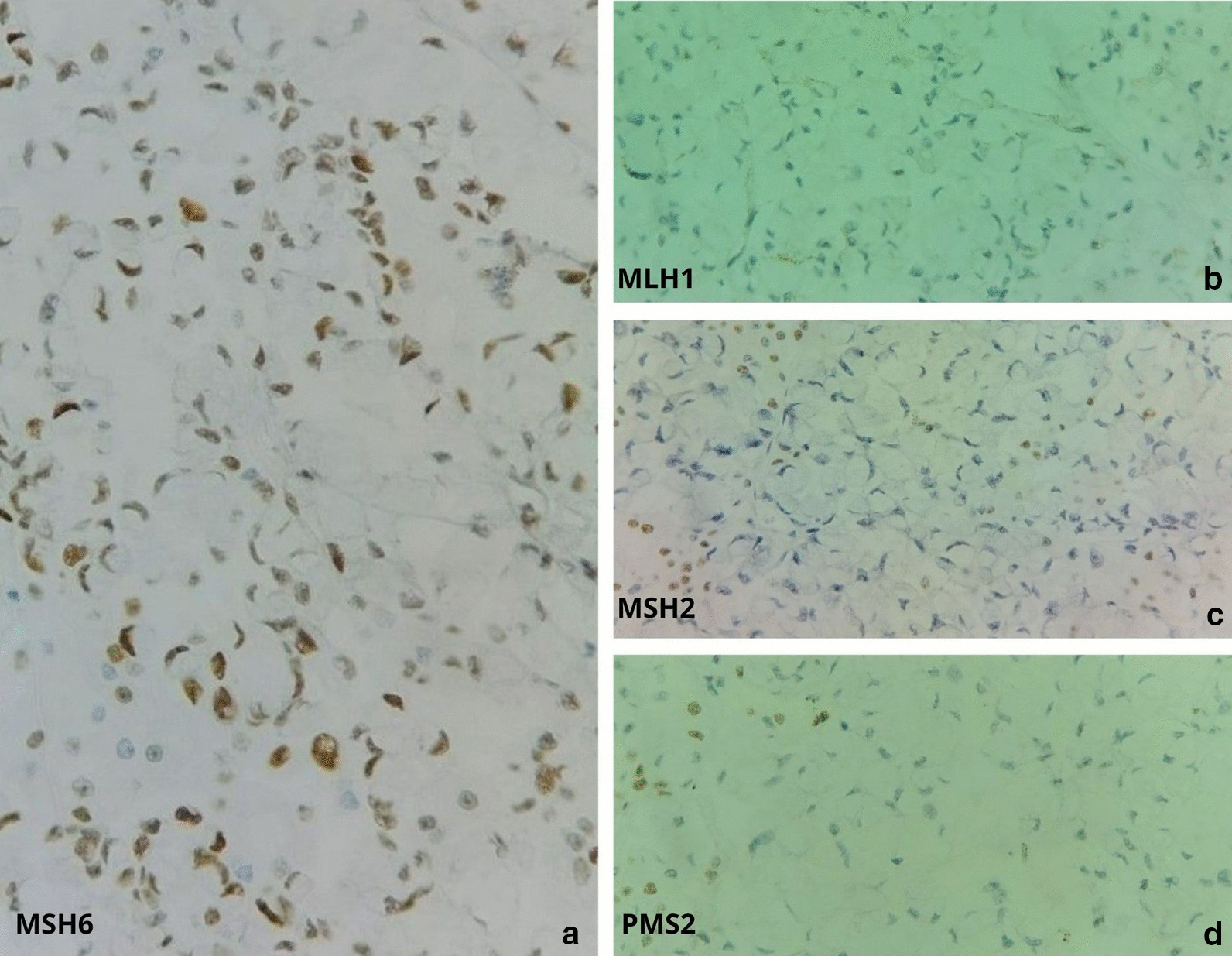
Immunohistochemistry for mismatch repair proteins, with only MSH6 showing nuclear expression (magnification: × 400), **a** MSH6, **b** MLH1, **c** MSH2, **d** PMS2

There was nuclear expression only of MMR protein MSH6, whereas the MMR proteins MLH1, MSH2 and PMS2 showed loss of the nuclear signal (Fig. 4). Therefore, the tumor was further analyzed molecularly using the ABI 310 DNA analyzer (Applied Biosystems, Foster City, CA, USA), which revealed a microsatellite stable (MSS) tumor. Molecular analysis showed mutations in TP53 and ERBB2, as determined by next-generation sequencing of AKT1, BRAF, EGFR, ERBB2, FOXL2, GNA11, GNAQ, KIT, KRAS, MET, NRAS, PDGFRA, PIK3CA, RET and TP53. The patient died a few months after surgical treatment.

## Discussion

### Primary rectal signet ring cell carcinoma

PSRCCR is a variant of colorectal cancer (CRC) with a rare, aggressive presentation and diagnosis at an advanced stage. It is more common in young adults than in older patients and has distinct molecular profiles, such as microsatellite instability and BRAF mutations [3–6]. Our case presented a microsatellite stable (MSS) tumor without BRAF activating mutations. A stomach origin was excluded in earlier examinations because this type of carcinoma usually presents as gastric carcinoma. Signet ring cells present a large amount of cytoplasmic mucin displacing the nuclei at the periphery. CT scan or radiologic imaging is part of the initial examination in patients with abdominal pain or other long-term symptoms of discomfort. The most common metastatic site is peritoneal seeding; there is a low incidence in the liver, lung and bone, with uncommon metastatic sites also in the vertebrae, extraocular muscles, bone marrow and skin [7–11]. Because of the rare occurrence, the clinical importance of PSRCCR is difficult to evaluate, but the poor prognosis is evident and accompanied by many synchronous and metachronous distant organ metastasis.

### Association with renal cell carcinoma

A few but strong studies have shown the occurrence of synchronous gastrointestinal and kidney tumors [12–14]. In their study of 101 patients, Steinhagen *et al.* emphasized the occurrence of both CRC and RCC, with a risk for additional malignancies [12]. RCC was observed at the median age of 67 years and was mainly of the clear cell type, with half of the cases found during the workup for CRC [12]. Our case also indicates an incidental finding of a kidney tumor during the colorectal imaging analysis. In their immunochemistry study, Steinhagen *et al.* found that an MMR protein was absent in one in ten patients with CRC, but the accompanying RCC retained all four proteins [12]. Our case showed nuclear loss of MLH1, MSH2 and PMS2 on immunohistochemistry, with DNA sequencing for microsatellite instability revealing that the tumor was MSS. A search of the PubMed library did not find a synchronous case of RSRCC with RCC, as presented in our patient.

### Tumor to tumor metastasis

The third rare event in our case was the tumor to tumor metastasis phenomenon. There must be at least two separate and histologically different neoplasms to confirm the feature. Why certain tumors are donors and some are recipients is a question that is open to debate. RCC is considered to be a well-documented recipient of metastasis by lung carcinomas, prostatic carcinomas, breast carcinomas, stomach adenocarcinomas, thyroid anaplastic carcinomas and uterine adenocarcinomas [15–18]. The vascularity or donor genetics of the recipient tumor might be the key to determining this metastatic pattern. Colorectal AC is reported to be able to metastasize to ovarian cystadenofibroma [17]. Sakai *et al.* reported a signet ring cell carcinoma of the stomach with metastasis into RCC after 6 years [18]. Our case presented metastasis in RCC of PSRCCR at initial diagnosis. Imaging techniques are unreliable on early detection of metastasis, and time is needed as well as histopathology examinations for confirmation.

## Conclusions

We present a case of signet ring cell carcinoma of rectum with metastasis to synchronous RCC and to the adrenal gland. RCC when diagnosed in multiple synchronous tumors should be examined carefully. Cancer to cancer metastasis as a rare phenomenon that needs to be considered in synchronous tumors. The paucity of reported cases indicates the need for advanced research in imaging methods for the detection of metastasis and new therapy approaches.

## Data Availability

Not available.

## Abbreviations

AC: Adenocarcinoma
CRC: Colorectal cancer
CK: Cytokeratin
PSRCCR: Primary signet ring cell carcinoma of the rectum
RCC: Renal cell carcinoma
